# Supplementary material for: Investigational Drugs for the Treatment of Postherpetic Neuralgia: Systematic Review of Randomized Controlled Trials
Source: Int J Mol Sci. 2023 Aug 20;24(16):12987. doi: 10.3390/ijms241612987 (PMC10455720; doi:10.3390/ijms241612987)
Supplement: Supplementary file 1 [file ijms-24-12987-s001.zip › ijms-2549861-supplementary.pdf]

Table S1. Information about secondary outcomes regarding efficacy of included studies.

Drug	Secondary pain outcomes	Other secondary outcomes (sleep, anxiety, etc.)
Olodanrigan<br>(EMA401) [24]	• NPSI total score (week 12) numerically in favor of placebo<br>• In EMA401, reductions in NPSI dimensional score (week 12) lower for all dimensions except for deep/pressing pain	Not measured
Crisugabalin<br>(HSK16149)<br>[13]	DPN patients (ADPS change from baseline)<br>• -1.23 for placebo<br>• -2.24 for 40 mg/d<br>• -2.16 for 80 mg/d; (p < 0.0001)<br>Responders rate versus placebo:<br>• ≥30%: 57.3% 40 mg, 51.4% 80 mg vs. 31.6%<br>• ≥50%: 32.0% 40 mg, 36.3% 80 mg vs. 18.1%	Not measured
Mirogabalin (DS-5565)<br>[14,15]	• BPI-SF: At week 14, all doses showed greater improvement from baseline versus placebo<br>• Improved PGIC score<br>• Long-term efficacy confirmed in open-label extension (flexible dosing 10 or 15 mg twice a day for 52 weeks)	• HADS and SF-MPQ (excluding VAS). At week 14, all doses showed greater improvement from baseline vs. placebo<br>• SF-36 subscales greater in mirogabalin groups: physical functioning, role—physical, bodily pain, general perception of health, vitality, social functioning, role—emotional, and mental health<br>• Improved sleep disturbance/somnolence. At week 14, all doses showed greater improvement from baseline vs. placebo
Pregabalin<br>(CR) [16]	• Weekly mean pain: LS mean difference from placebo of 1.00; (p < 0.0001)	• Improved significantly HADS<br>• Sleep interference (MOS-SS): LS mean difference from placebo of 0.85; (p < 0.0001)
Pregabalin [17]	Proportion of patients "very much improved" or "much improved":<br>• CGIC (50.0% vs. 22.7%, p < 0.0001)<br>• PGIC (40.4% vs. 18.6%, p < 0.0001)	• SF-MPQ VAS and SF-MPQ PPI versus placebo (all p < 0.001)<br>• Improved sleep interference, quality and disturbance in MOS-SS
Pregabalin<br>(YHD1119)<br>[18]	• TD mean DPRS score not statistically significant (p > 0.05 for all visits)<br>• Between-group difference in mean DPRS score at the end of treatment: LS mean difference: 0.08 (95% CI -0.36 to 0.52; p non-inferiority <0.0001)	Not measured
Funapide (TV-45070; XEN402; XPF-002)<br>[19]	• DSIS, NPSI, and PGIC not significant.<br>• Placebo LS mean change in DSIS −0.84<br>• TV-45070 LS mean change in DSIS −0.81 (p = 0.8801)<br>• Mean change in NPSI score:<br>• −6.1 on placebo<br>• −6.7 on TV-45070<br>• Placebo response reduced in R1150W carriers	Not measured

Lidocaine [20]	Tramadol used per patient:<br>• Control group, 0.35 ± 0.43 g<br>• Lidocaine 0.16 ± 0.29 g ($p < 0.05$)	• No difference in HADS (time of admission nor day of discharge.<br>• Average hospital stay:<br>• Control 8.8 ± 0.7 days<br>• Lidocaine 6.8 ± 0.6 days ($p < 0.0001$)
TRK-700 [25]	No publications available. Last updated: July 2017	
LX9211 [26]	No results published. Last updated: March 28, 2023. No other secondary outcomes in the register	
LAT8881 (AOD9604)<br>[27]	No TD in any secondary outcomes:<br>• NPRS score after the first and last dose<br>• NPRS scores after 1, 2 and 3 weeks of treatment, maximum change in mean NPRS<br>• Change in pain characteristics and intensity (SF-MPQ)<br>• Change in neuropathic pain symptoms (NPSI)	No TD in any secondary outcomes:<br>• Change in emotional functioning (Beck Depression inventory II)<br>• Change in functioning (BPI)
SR419 [28]	No results published. Other outcome: Within-subject difference between the two treatment periods in the weekly average of daily sleep interference scores at the last week	
Esketamine [29]	Not finished yet. At the end, a 7 Tesla scan will be performed on the right-hand patients with herpes on the body to explore the effect of ketamine on the brain network	
Sustained-release tramadol (NZ-687) [21]	• NRS value decreased in tramadol group for 1–5 weeks in the open-label phase (all $p < 0.0001$)<br>• SF-36 score for bodily pain increased significantly from 42.7 ± 16.0 to 52.5 ± 17.5, mean change of 9.8 ± 16.6 ($p < 0.0001$)	• Significant improvements (mean change ≥2) in SF-36 domains for physical functioning, vitality, and mental health (all $p < 0.05$)
Oxycodone [22]	No significant difference between oxycodone or vehicle patch in:<br>• Change of daily average pain<br>• Change from baseline worst pain<br>• NPSI	Not measured
Hydromorphone (PCA) [23]	• Frequency of breakthrough pain lower versus control 1 week and 4 weeks after treatment (all $p < 0.001$)	• PSQI 4 and 12 weeks after treatment, (all $p < 0.001$)<br>• SF-MPQ at 1, 4 and 12 weeks (all $p < 0.001$)
Fulranumab<br>(JNJ-42160443) [30]	• No differences in worst pain in the past 24 h<br>• No differences in the NPSI total scores<br>• Improvement in pain relief subscale only for 3 mg ($P = 0.04$) in the BPI-SF scale	• 55% for both placebo and fulranumab groups reported their status as "not changed" in PGIC

NPSI: Neuropathic Pain Symptom Inventory; DPN: Diabetic Peripheral Neuropathy; ADPS: Average Daily Pain Score; BPI-SF: Brief Pain Inventory Short Form; PGIC: Patients' Global Impression of Change; HADS: Hospital Anxiety and Depression Scale (HADS); SF-MPQ: Short-Form McGill Pain Questionnaire; VAS: Visual Analogue Scale; SF-36: 36-Item Short Form Survey; CR: Controlled Release; LS: Least Squares; MOS-SS: Medical Outcomes Study Sleep Scale; CGIC: Clinical Global Impression of Change; PPI: Present Pain Intensity; TD: Treatment Difference; DPRS: Daily Pain Rating Score; CI: Confidence Interval; DSIS: Daily Sleep Interference Score; NPRS: Numerical Pain Rating Score; BPI: Brief Pain Inventory; NRS: Numerical Rating Score; PSQI: Pittsburgh Sleep Quality Index.

Table S2. Information about serious adverse events reported in the included studies.

Drug	Serious Adverse Events (SAEs)
Olodanrigan (EMA401) [24]	No SAEs in EMA401 25 mg, whereas there were 3 cases (7%) in EMA401 100 mg (lower RTI, traumatic hematoma and ECG ST elevation) and 5 (7%) in placebo (non-cardiac chest pain, back pain, OA, CNS lymphoma and lumbar radiculopathy)
Crisugabalin (HSK16149) [13]	No DR SAEs or deaths occurred in this study
Mirogabalin (DS-5565) [14,15]	Fifteen patients had an SAE: 5 in placebo, 5 in DS-5565 15 mg/day, 2 in DS-5565 20 mg/day, and 3 in DS-5565 30 mg/day. Two patients in DS-5565 30 mg/day and one patient in placebo had increased blood creatine phosphokinase (transient). Others included Meniere disease, AMI, and radius fracture in placebo; pneumonia and two fractures in DS-5565 15 mg/day; erectile dysfunction and two fractures in DS-5565 20 mg/day; and memory impairment, altered state of consciousness, cerumen impaction, and ECG change in DS-5565 30 mg/day
Pregabalin (CR) [16]	A total of 10 patients experienced an SAE: 3 in placebo (1.5%) and 7 in pregabalin (3.4%). SAE consisted of thrombocytopenia, bronchopneumonia and CVA for placebo, and anaemia of chronic disease, cardiac failure, acute sinusitis, perirectal abscess, back pain, sciatica and renal mass for pregabalin (each corresponding to one patient). A total of 9 patients discontinued their treatment because of an AE: 6 in placebo (2.9%) and 3 in pregabalin (1.4%). Two deaths were reported (one due to severe acute respiratory syndrome and the other one due to severe CVA, both in placebo)
Pregabalin [17]	Two SAEs (1.8%) were reported in pregabalin (RTI and cerebral ischemia), with neither of these being DR
Pregabalin (YHD1119) [18]	No DR SAEs or deaths occurred in this study. Incidence of DR AEs leading to discontinuation was low in both groups (IR pregabalin: 1.1%; SR pregabalin: 2.7%)
Funapide (TV-45070; XEN402; XPF-002) [19]	There were two SAEs, neither considered DR: tooth abscess occurred during placebo treatment and the worsening of CAD occurred on the first day of TV-45070 treatment. The patient withdrawn from the study had previously undergone stenting and cardiac catheterization and was managed with angioplasty
Lidocaine [20]	No DR SAEs or deaths occurred in this study<br>Patients' vital signs, ECG and other indicators were normal
TRK-700 [25]	No clinical results published
LX9211 [26]	No SAEs or deaths occurred in Phase I studies. One participant (50/5-mg) discontinued treatment on day 6 because of AEs (angioedema, dermatitis allergic, and urticaria). They were mild and possibly DR
LAT8881 (AOD9604) [27]	No DR SAEs or deaths occurred in this study
SR419 [28]	No clinical results published

Esketamine [29]	None of the AEs was serious
SR tramadol (NZ-687) [21]	Two patients experienced SAEs. One patient in the OLP experienced pneumonia (moderate in severity and non-DR) which resolved after discontinuation of 200 mg/day tramadol and symptomatic treatment. Congestive heart failure was reported as an SAE (non-considered DR) in one patient in 400 mg/day tramadol, which resolved after discontinuation and treatment).
Oxycodone [22]	No AEs commonly associated with opioids were reported<br>No DR SAEs or deaths occurred in this study
Hydromorphone (PCA) [23]	No AEs commonly associated with opioids were reported<br>No DR SAEs or deaths occurred in this study
Fulranumab<br>(JNJ-42160443)<br>[30]	During the combined DB efficacy and extension phases, 7 patients experienced SAEs:<br>• 10% in placebo<br>• 8% in 1 mg JNJ-42160443<br>• 21% in 10 mg JNJ-42160443<br>No deaths were reported

AMI: Acute Myocardial Infarction; CAD: Coronary Artery Disease; CNS: Central Nervous System; CVA: Cerebrovascular Accident; DB: double-blind; DR: Drug Related; ECG: Electrocardiogram; IR: Immediate Release; OA: Osteoarthritis; OLP: Open-Label Phase; RTI: Respiratory Tract Infection; SAE: Serious Adverse Event; SR: Sustained Release
